# Understanding Glioblastoma Signaling, Heterogeneity, Invasiveness, and Drug Delivery Barriers

**DOI:** 10.3390/ijms241814256

**Published:** 2023-09-19

**Authors:** Nadin Rabah, Fatima-Ezzahra Ait Mohand, Nataly Kravchenko-Balasha

**Affiliations:** The Institute of Biomedical and Oral Research, Hebrew University of Jerusalem, Jerusalem 91120, Israel; nadin.rabah@mail.huji.ac.il (N.R.); fatima-e.aitmohand@mail.huji.ac.il (F.-E.A.M.)

**Keywords:** glioblastoma, recurrence, heterogeneity, invasion, blood–brain barrier (BBB), targeted therapy, GBM signaling

## Abstract

The most prevalent and aggressive type of brain cancer, namely, glioblastoma (GBM), is characterized by intra- and inter-tumor heterogeneity and strong spreading capacity, which makes treatment ineffective. A true therapeutic answer is still in its infancy despite various studies that have made significant progress toward understanding the mechanisms behind GBM recurrence and its resistance. The primary causes of GBM recurrence are attributed to the heterogeneity and diffusive nature; therefore, monitoring the tumor’s heterogeneity and spreading may offer a set of therapeutic targets that could improve the clinical management of GBM and prevent tumor relapse. Additionally, the blood–brain barrier (BBB)-related poor drug delivery that prevents effective drug concentrations within the tumor is discussed. With a primary emphasis on signaling heterogeneity, tumor infiltration, and computational modeling of GBM, this review covers typical therapeutic difficulties and factors contributing to drug resistance development and discusses potential therapeutic approaches.

## 1. Introduction

Glioma is a term used to describe a neuroepithelial tumor originating from glial cells, which are the most common type of supporting cells in the CNS. One of the deadliest types of human cancer, namely, glioblastoma multiforme (GBM), is classified as a WHO grade IV brain tumor. A combination of surgical resection, radiation therapy, and chemotherapy are the current accepted treatments for GBM [1].

Surgery to remove a GBM tumor carries a significant danger for the patient since it frequently invades vital brain regions. Following surgery, patients receive concurrent Temozolomide (TMZ) and radiation therapy. Unfortunately, these methods only slightly improve the prognosis for GBM patients, with a median survival of 14–15 months and a 5-year survival rate of about 10% [2,3].

**Figure 1:**
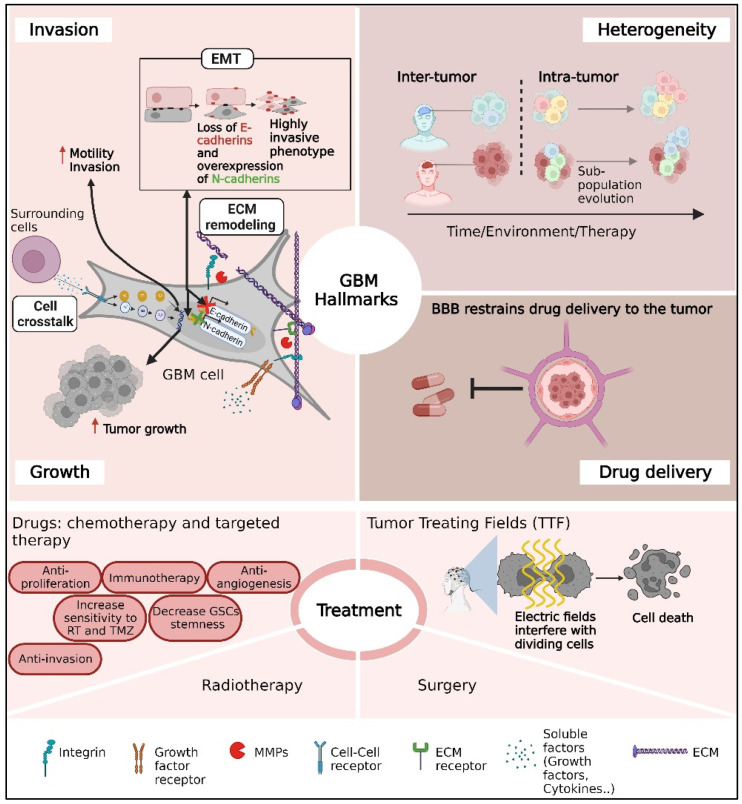
A schematic overview of the main challenges for glioblastoma (GBM) clinical care mentioned in this review. The challenges include the infiltrative nature of GBM tumors, substantial heterogeneity between patients or cells inside the tumor, drug delivery through the BBB, and therapeutic limitations.

In this review, we highlight three major problems in GBM treatment: tumor heterogeneity, GBM infiltration, and the blood–brain barrier (BBB) (Figure 1).

Significant efforts have been made to subtype GBM in order to address the problem of inter-tumor heterogeneity, including methylation (six subtypes), transcriptomic subtyping (proneural, neural, classical, and mesenchymal), and the World Health Organization definition (IDH wild type, IDH mutated, NOS) [4,5]. However, when protein–protein expression patterns are estimated over a large population of GBM tumors in proteome variability studies, they reveal even more variations between GBM patients, resulting in dozens of subgroups [6]. Furthermore, tumor cell variability (intra-tumor), which is often dynamic and develops over time, adds another layer of complexity to tumor classification [7,8,9].

Another clinical difficulty is the highly diffuse nature of GBM. Even when a tumor is successfully removed, followed by a combination of radiotherapy and chemotherapy, GBM recurs. Tumor relapse is frequently caused by highly infiltrative cells that penetrate the brain and are not recognized during surgery [10]. Enhanced cellular infiltration is caused by several processes, including signaling pathways involved in EMT (epithelial–mesenchymal transition), ECM remodeling, and increased spreading due to cell–cell communication inside GBM [11].

The anatomical position of GBM provides a further significant barrier to treatment, as GBM cells are inaccessible to most systemically administered medicines [1].

Taken as a whole, the review expands on these issues (Figure 1) and recent initiatives to solve them, whether through novel treatment options, recent research, or technological improvements. Other important topics, such as immune checkpoint treatment, CAR T-based immunotherapy, oncolytic viral medicines, gene and thermo-therapies, and tailored neoantigen-based vaccinations, were recently reviewed elsewhere [12,13,14].

## 2. Current Glioblastoma Treatments

The degree of GBM resection is a crucial element in patients’ survival. The gross total resection (GTR) was found to be favorably connected to survival time [15,16]. However, achieving GTR and removing the entire tumor is rare. An exact high-level determination of the tumor margins is required for a successful GTR. Surgical removal becomes extremely difficult when a tumor is located in an unresectable brain area or next to an area responsible for neurological function. Furthermore, due to the invasive nature of GBM, tumor cells infiltrate alone or in small groups into the healthy area, making identifying tumor margins and, ultimately, resecting the entire tumor without harming vital parts impossible [1]. As a result, post- or pre-surgical treatments and non-surgical treatment modalities are required to prevent tumor recurrence.

The following sections contain a list of available anti-GBM treatments and a discussion of recent developments in GBM research that aim to increase the field’s current understanding of the disease.

### 2.1. Temozolomide (TMZ) Therapy

Temozolomide (TMZ) is a first-line chemotherapeutic drug used to treat post-surgical GBM [17]. It is an oral alkylating drug that produces DNA adducts by methylating purine bases in DNA [18]. Its chemical nature makes it a preferred chemotherapeutic agent for GBM because of its stability at stomach acidic pH and ability to pass the blood–brain barrier (BBB) [19].

The treatment regimen has mostly stayed the same since a clinical trial in 2005 that confirmed the efficacy of adding TMZ to RT in a group of patients with newly diagnosed GBM [20]. Unfortunately, despite its contribution to patient survival (a 37% increase in survival at a median follow-up of 28 months) [20], the development of TMZ resistance and thus, the incidence of tumor recurrence, remains very high [21].

TMZ resistance can be developed through a variety of mechanisms [19,22], including MGMT (O6 Methylguanine DNA Methyltransferase) overexpression [23]. MGMT is a DNA repair protein that removes the methyl group from O6-methylguanine to prevent DNA alkylation [24].

Another mechanism shows that repeated TMZ treatment of GBM cells may transform non-GSCs (GBM stem cells) into new GSCs [25], enhancing their potential to self-renew, proliferate indefinitely, and differentiate into many lineages [26].

TMZ can also cause thrombocytopenia in patients, which is caused by TMZ-dependent DNA damage in healthy cells [27,28].

Furthermore, recent research has shown that TMZ treatment may contribute to tumor progression by promoting tumor invasion and EMT (epithelial–mesenchymal transition). Kubelt et al., for example, demonstrated that TMZ therapy enhances mRNA expression of many EMT markers in T98G glioma cells in vitro, including Vimentin, TGF-β, and Fibronectin [29]. Kochanowski et al. revealed that Cx43 signaling promotes GBM cell invasiveness in both MGMThigh (T98G) and MGMTlow (U87) populations [30]. The “GO OR GROW” phenomenon, which was reported in slow-dividing but spreading cells, was also detected in IDH-mutant astrocytoma and IDH-WT GBM cells after TMZ treatment. These cells reduced their proliferation rate significantly in response to TMZ but moved 2–3 times faster [31].

### 2.2. Radiotherapy (RT)

Radiotherapy is recommended either alone or in conjunction with TMZ after a surgical GBM resection. Despite the long history of radiation therapy in GBM therapy, ongoing debates exist about its usefulness as a standard treatment technique and its involvement in GBM recurrence.

The major mechanism through which RT causes cell death is DNA damage. The manner in which cells respond to this injury, however, is determined by cell-intrinsic and microenvironmental factors [32]. Current clinical practice and research show that radiotherapy is less effective in some brain cancers than others [33,34]. Glioblastomas are among the most radio-resistant aggressive forms of cancer [35]. Several efforts were made to identify biomarkers to determine and select patients with radio-sensitive tumors [36].

For example, in a study that ranked the radiosensitivity of 40 human cell lines based on survival components, three glioblastoma cell lines, namely, U87, U251, and T98G, were found to be the most radio-resistant cell line models [34]. The radiosensitivity was found to be mainely associated with p53 status and the expression of the ATM gene, which plays a critical role in regulating the DNA damage response [33]. A similar pattern was reported in several in vivo models [37,38,39]. For instance, the combined effects of radiation and the ATM kinase inhibitor (KU-60019) significantly increased mice survival by 2-3-fold compared with controls [38]. Furthermore, the mutant p53 group was substantially more radiosensitive to KU-60019 than p53 WT [39,40].

MDM2-mediated p53 suppression is another axis that was found to be disturbed in at least 25% of primary and 60% of secondary GBM. The MDM2/p53 axis was reported to reduce the efficacy of RT in the treatment of cancer [41]. Thus, inhibiting the MDM2/X-p53 interaction is recognized as a potential anti-cancer strategy, including in the treatment of glioblastoma [42].

Radio-resistance may also arise from the cancer stem cell (CSC) subpopulation, hypoxia, and the increased expression of DNA repair pathways [43]. Moreover, irradiation, similarly to TMZ, can promote cancer progression. For instance, it was observed that primary malignancies treated with brain radiation had a seven-fold greater chance of developing secondary CNS tumors [2].

Besides the radio-resistance mechanisms, RT was found to induce enhanced invasion and cell “escaping” from the primary tumor [44], which is one of the main reasons for tumor relapse [45], especially at the post-surgical margins, which occurs at rates of up to 90% [46].

Cells that survive the lethal effects of RT are frequently aggressive, multiply more quickly, and have improved migratory and invasion capabilities [47]. It was found that modest dosages of 5-8 Gy, which cannot be increased due to safety reasons [48], enhance tumor cell invasion [49,50]. It is yet not fully clear why low radiation exposures increase cell migration. Several studies found that RT changes the expression and functional activities of adhesion molecules [51], such as the upregulation of α_v_ β_3_ integrin expression following RT [52]. Another study found that RT can activate Src-dependent EGFR, which activates the p38/Akt and PI3K/Akt signaling pathways, resulting in enhanced MMP-2 secretion and invasiveness of PTEN mutant glioma cells [53].

### 2.3. Tumor-Treating Fields (TTFs)

A TTF is a relatively recent non-invasive therapeutic approach that involves administering alternating, low-intensity, intermediate-frequency electric fields (100–300 kHz) to tumor cells [54]. The TTF devices comprise nine insulated electrodes placed on the patient’s scalp to deliver electric fields that aim to disrupt cell division and reduce tumor growth [55]. TTFs, in particular, interrupt the normal polymerization–depolymerization process of microtubules during mitosis. In terms of cell morphological changes in response to therapy, their effect is comparable with that of Taxol [55].

The US Food and Drug Administration (FDA) approved a TTF device in 2011 to treat recurrent or resistant GBM. Together with the National Comprehensive Cancer Network (NCCN), they recently approved the TTF device as an adjuvant treatment for newly diagnosed GBM patients who have completed standard-of-care surgery and chemoradiation [56]. However, a TTF is not regarded as a “standard of treatment” for GBM patients, owing to incomplete clinical trials that lacked a placebo-control “sham” device and demonstrated poor safety, in addition to the device’s high cost [56].

The poor response of GBM tumors to existing treatments spurred researchers worldwide to define the mechanisms and GBM phenotypes that cause therapeutic resistance. The primary mechanisms are listed below.

## 3. Infiltration and Invasion of GBM Cells

The invasiveness of GBM is a major reason for therapeutic failure. Cancer cells that remain after surgery or treatment frequently create a new mass within 2–3 cm of the original lesion [57]. While other aggressive cancers spread to organs via the circulatory or lymphatic systems, high-grade glioma cells migrate actively through two types of extracellular space in the brain: (1) perivascular space near blood vessels and (2) space between neurons and glial cells that make up the brain parenchyma and white matter fiber tracts [58].

### 3.1. Epithelial–Mesenchymal Transition (EMT)

The EMT is a critical mechanism in physiological and pathological processes, such as embryogenesis, wound healing, and cancer development, enabling cells to transit from an epithelial to a mesenchymal state [59]. The literature defines three distinct EMT subtypes, each of which occurs in different biological circumstances and has a range of functional outcomes [60,61].

Normal tissue homeostasis, including embryonic development, is influenced by type 1 EMT, while type 2 EMT occurs in wound healing, organ fibrosis, and tissue regeneration [61]. Type 3 EMT, on the other hand, is associated with neoplastic cells that have undergone genetic and epigenetic changes, boosting tumor-initiating and metastatic potential, as well as resistance to different treatment regimens [62].

EMT causes epithelial cells to separate when cells lose their apical–basal polarity and connections through tight junctions. In other words, epithelial cells lose their ability to adhere and instead develop a fibroblast-like shape and enhanced mobility during EMT. EMT simultaneously promotes the expression of mesenchymal marker proteins and aids in developing mesenchymal characteristics and attachment to the extracellular matrix (ECM) [63].

Type 3 EMT plays a dominant role in GBM tumors [64], where it is associated with poor prognosis, tumors’ immune escape [65], and invasive behavior of GBMs, coupled with autophagy, which is a cellular process involved in the degradation of cytosolic protein aggregates, supporting cellular and organismal homeostasis [66]. GBM cells become more capable of migrating and invading through blood vessels and basement membranes as they approach the mesenchymal state. Since GBM cells do not originate from epithelial cells, the EMT process in GBM is referred to as EMT-like.

N-cadherin overexpression and E-cadherin loss are two of the most noticeable characteristics of EMT [57,67]. The loss of E-cadherin causes changes in cell adhesion and increases cell spreading since it is an epithelial marker that prevents epithelial cells from separating from the parent tissue [68]. On the other hand, it was demonstrated that brain tumor invasion is increased by overexpressing N-cadherin, which is a transmembrane adhesion protein [68,69].

Eukaryotic Translation Elongation Factor 1 Delta (EEF1D) and Calponin and LIM Domain Containing 2 (MICAL2) are two further examples of EMT-related markers. It was demonstrated that EEF1D increases glioma proliferation and invasion via modifying the EMT process and that blocking EEF1D might reverse the EMT properties of glioma cells, decreasing cell growth and tumor progression [70]. In addition, a TGF-B/p-Smad2/EMT-like signaling pathway was discovered to increase GBM growth and invasion [71].

Recently, it was discovered that the radioresistant characteristics of some gliomas and GBMs are related to the overexpression of EMT genes, such as *ACTN1*, *CCND1*, *HCLS1*, *ITGB5*, *PFN2*, *PTPRC*, *RAB13*, and *WAS* [72]. Irradiation was also shown to increase the expression of Vimentin and other EMT-associated proteins, which may play a role in cell migration [46,73]. Furthermore, it was proposed that Vimentin can cause EMT movement by upregulating N-cadherin and downregulating E-cadherin [74].

### 3.2. The Extracellular Matrix (ECM) Has a Role in Cell Invasion

GBM cells invade by undergoing many biological changes that ultimately result in remodeling the cellular cytoskeleton and the surrounding extracellular matrix (ECM). Even though the ECM is a physical barrier that GBM cells must overcome, it also provides ligands, such as integrins [58], to which the tumor cells can anchor to propel themselves forward and carry out the mesenchymal migration [75]. It was shown, for example, that RT can alter the ECM by upregulating integrins, facilitating GBM infiltration [76]. 

Cell adhesion, migration, and cell fate decisions are significantly influenced by the molecular and physical properties of the ECM. Normal brain tissue’s ECM comprises hyaluronan, proteoglycans, and tenascin-C, and it lacks fibrillar collagens’ capacity to create stiff ECM structures [77]. In contrast, the ECM in GBM is associated with a significant increase in basement membrane constituents like collagens and laminin [10,78].

Laminin expression and peri-tumor collagen production were both found to be upregulated at the invasive edge of GBMs [79], favoring cancer cell invasion and metastasis in different cancer models [80,81].

Collagen type I, which acts as a structural framework for cells and other scaffold proteins [82], is one of the most abundant connective tissue components. Collagen I may be prevalent in some tumor types, like lung carcinoma [83]; however, in GBM [84], collagen types IV and VI were shown to be more essential for GBM tumor growth, vascularization, and migration [85,86,87]. Thus, ECM elements, including collagens, were proposed as potential therapeutic targets for GBM [47,78,88].

## 4. Signal Transduction Pathways and Targeted Therapies

The primary regulatory pathways that support GBM tumor invasion, survival, drug resistance, and anti-apoptotic characteristics are listed below.

### 4.1. Epidermal Growth Factor Receptor (EGFR)

**Figure 2:**
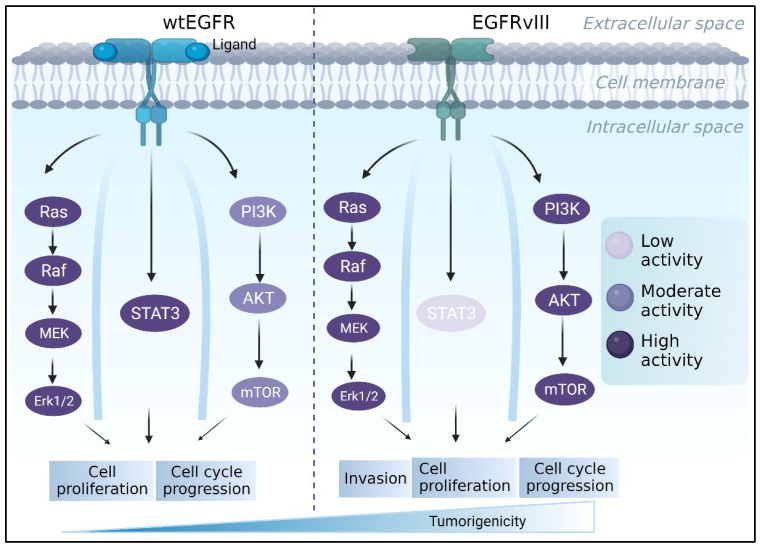
The EGFR/EGFRvIII pathway contributes to the progression of GBM. While PI3K and RAS/MAPK activation is stronger in EGFRvIII-expressing cells, EGFRwt commonly stimulates MAPK and STAT3, encouraging tumor growth. Meanwhile, EGFRvIII is constitutively activated and primarily initiates the PI3K pathway, which is involved in tumor invasion and survival.

The epidermal growth factor receptor (EGFR) has been designated as a “signature molecule” for glioblastoma [89,90]. EGFR is a tyrosine kinase receptor that regulates multiple signaling pathways involved in cell proliferation, differentiation, and migration [91], including PI3K/AKT, RAS/RAF/MEK/MAPK, and STAT cascades [92,93,94] (Figure 2). The *EGFR* gene amplification is common in all gliomas, accounting for 40 to 50 percent of primary GBMs [95].

*EGFRvIII* is a mutant EGF receptor that lacks the extracellular ligand-binding domain (exons 2–7 deletion) and is constitutively active in 50-60% of *EGFR*-amplified GBMs [96]. GBM tumors that express wtEGFR frequently express the EGFR lesion [97,98,99]. EGFRvIII expression, on the other hand, is uncommon in the absence of wtEGFR amplification [98,100]. The difference between the two receptors also appears in their activated downstream pathways. For instance, it was reported that EGFRvIII cells had substantially higher PI3K activity than did cells with wtEGFR [101]. EGFRvIII, unlike wtEGFR, does not appear to activate the STAT3 pathway via direct phosphorylation [102]. Furthermore, a study found that U87MG GBM cells engineered to express EGFRvIII increased RAS activity twice as much as parental cells [103] (Figure 2). EGFRvIII was regularly found to be more tumorigenic than wtEGFR [104,105,106]. Nude mice injected with U87MG GBM cell lines harboring EGFRvIII, for example, form tumors faster than parental U87MG cells or cells expressing wtEGFR [107]. Furthermore, NR6 murine fibroblasts harboring EGFRvIII demonstrated increased motility, while U87 MG cells transfected with EGFRvIII demonstrated increased migration and invasion [108,109]. This phenomenon was compatible with clinical observations [110].

Recently, it was demonstrated that murine astrocytes expressing EGFRvIII were significantly less adhesive by reducing their focal adhesion size and number and displayed enhanced migration compared with cells bearing mutations in *Ink4a* or *PTEN* [111].

### 4.2. Vascular Endothelial Growth Factor Receptor (VEGFR)

Three Receptor Tyrosine Kinases (RTKs) are members of the Vascular Endothelial Growth Factor Receptors family (VEGFR1-3). It was shown to influence vasculogenesis and angiogenesis [112,113]. Because of unregulated angiogenesis and vascularization in GBM tumors, VEGFR2 signaling is disrupted, resulting in uncontrolled survival, migration, and vessel permeability [114]. Furthermore, the surrounding hypoxic microenvironment of glioblastoma induces VEGFR signaling, allowing the tumors to compensate for the hypoxia [115]. In addition to its angiogenic involvement in glioblastoma, a few studies revealed that VEGFR may be involved in the irradiation-dependent motility and proliferation of GBM cells [116].

### 4.3. Fibroblast Growth Factor Receptor (FGFR)

The Fibroblast Growth Factor Receptor family consists of four receptors, namely, FGFR 1-4. They control essential biological processes involved in development, including differentiation and proliferation, as well as CNS growth and tissue repair [117,118].

Although FGFR genetic mutations are regarded as infrequent in glioblastoma, FGFR overexpression in astrocytes may contribute to malignant transformation and GBM progression [119]. FGFR3 and FGFR4 were shown to be upregulated in invasive GBM cells [120]. Furthermore, *FGRF1* and *FGFR3* gene fusions with transforming acidic coiled-coil genes (*FGFR-TACC*) were discovered in GBM, leading to constitutive receptor activation and aneuploidy [121].

### 4.4. Platelet-Derived Growth Factor Receptor (PDGFR)

The platelet-derived growth factor receptor family includes two receptors, namely, PDGFRα and PDGFRβ, which are required for tissue development during embryogenesis. PDGF mutations were implicated in GBM development and metastasis [122]. The two receptors contribute to the progression of GBM in separate ways. While PDGFRα, which is the second most amplified receptor in glioblastoma after EGFR, is primarily upregulated in the proneural subtype, PDGFRβ is preferentially expressed in glioma stem cells (GSCs), where it regulates the level of stem cell markers, like SOX2 [4,123]. Furthermore, PDGFRα mutations are strongly linked to the occurrence and poor prognosis of GBMs [124].

### 4.5. Hepatocyte Growth Factor Receptor (HGFR)

The hepatocyte growth factor receptor, also known as c-MET, is recognized to play an essential role in the interaction of mesenchymal and epithelial cells throughout embryogenesis and tissue homeostasis. HGFR-activating ligands are abundantly produced by GBM cells, resulting in significant activation of PI3K, STAT3, and RAS pathways [125,126]. *HGFR* amplification was found in 1.6-4 percent of GBM samples, and its presence was linked to a poor prognosis [98,127].

### 4.6. Insulin-like Growth Factor Receptor 1 (IGF1R)

The insulin-like growth factor receptor 1 is a member of the IGF receptor family, which is known to play important functions in prenatal and postnatal development [128]. While IGF1R expression in normal tissue regulates cell proliferation and differentiation during brain development, its upregulation in GBMs is associated with enhanced activation of PI3K/Akt and MAPK pathways, leading to neoplastic transformation, TMZ resistance, and poor patient survival [129,130].

### 4.7. Discoidin Domain Receptors (DDRs)

Triple-helical collagen activates the discoidin domain receptors, namely, DDR1 and DDR2, in a delayed and sustained manner [131,132]. DDRs play a vital role in embryonic development by regulating a variety of processes, including proliferation, migration, adhesion, and ECM remodeling [133]. GBMs are distinguished by a high amount of collagens, which alters DDR signaling and ECM stiffness, thereby influencing tumor progression [86]. DDR1 overexpression in GBM cells is associated with increased migration and invasion [134] and poor clinical outcomes [135]. In *DDR2*-mutated GBM, increased cell–ECM interactions were connected to tumor invasion [136].

### 4.8. Tyrosine Kinase with Immunoglobulin-like and EGF-like Domains (Tie) Receptors

Tie1 and Tie2 are members of the Tie receptor family. They are required for angiogenic vascular remodeling during embryogenesis and to control lymphangiogenic responses [137]. Tie2 expression in nonvascular glioma compartments correlates with glioma development and grade [138,139]. Following this finding, it was suggested that Tie2 signaling may facilitate cross-talk in the tumor microenvironment (TME) between glioma cells and vascular endothelial cells [139]. Ang-2, which is its agonist, was shown to be overexpressed in GBMs, where it is related to Tie2 activation, increased invasion, and reduced VEGF inhibition [140,141,142].

### 4.9. RTK Downstream Signaling

Ras/MAPK, PI3K/AKT/PTEN, FAK/SRC, and DNA repair signaling cascades are commonly disturbed in GBM [139,143].

Despite lacking RAS mutations, the protein is significantly activated in glioblastoma due to Receptor Tyrosine Kinase (RTK) activation, such as EGFR [144]. The upregulation of H-Ras or K-Ras was found to induce astrocyte transformation into malignant and infiltrating gliomas [145,146]. In addition to contributing to gliomagenesis, the RAS/MAPK signaling pathway is also involved in tumor maintenance [147].

The PI3K/AKT/PTEN and FAK/SRC pathways, which control cell proliferation, invasion, metastasis, and metabolism, are additional critical signaling pathways in GBM [148,149,150]. PI3K/AKT/PTEN is often active in GBMs due to PTEN and PI3K mutations or Akt amplifications [151], and it plays an important role in the development and progression of gliomas [152]. The upregulation of FAK/SRC induces the invasion and metastasis of GBM malignancies. Furthermore, SRC was linked to GBM maintenance via TME inflammation and metabolic rewiring [153].

DNA damage response [154,155,156] is one of the fundamental mechanisms driving radiation or chemotherapy resistance. It was shown that EGFR may activate ATM, which is one of the main regulators and effectors of the DNA-damage-activated checkpoint system, which causes radiation resistance in EGFRvIII tumor cells [157,158].

### 4.10. Targeted Therapy

**Figure 3:**
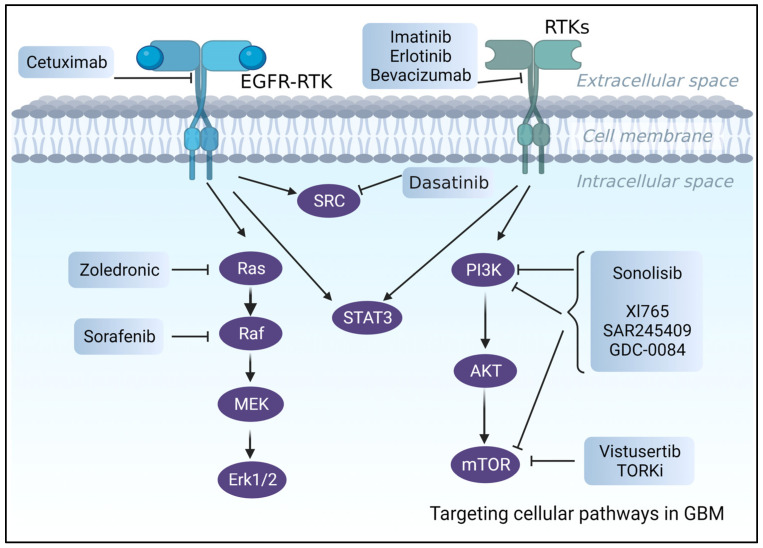
An illustration of the suggested pharmacological treatments and biological targets for the treatment of GBM.

Although significant progress has been made recently in understanding the molecular mechanisms underlying the malignancy of glioblastomas [159,160,161], which also resulted in the identification and validation of prognostic and predictive biomarkers [162,163], targeted therapies have so far demonstrated only modest clinical trial efficacy [160] (Figure 3).

Even though more than >70% of GBM patients have overexpressed RTK [164], such as EGFR and PDGFRA with active downstream pathways (e.g., PI3K/AKT/mTOR) [165], clinical trials including RTK inhibitors, such as Cetuximab, an FDA-approved anti-EGFR [166] antibody, or Imatinib (anti-PDGFRA inhibitor), have failed. Poor BBB permeability, intertumor heterogeneity, unfavorable side effects, and toxicities were the leading causes of these failures [160,167]. Despite promising results in preclinical investigations [168], erlotinib, which is another EGFR tyrosine kinase inhibitor (TKI), exhibited poor efficacy and unacceptable side effects [169] in phase 2 clinical trials of newly diagnosed or recurrent GBM patients.

Other examples are VEGF inhibitors [167,170], such as Bevacizumab, which is an FDA-approved, humanized monoclonal antibody that failed clinical trials due to low overall patient survival [170].

Several studies were undertaken to evaluate the idea of using inhibitors of downstream pathways (PTEN/PI3K/AKT/mTOR) rather than targeting upstream receptors [160,171]. However, despite promising results in in vitro and in vivo preclinical studies, PI3K and RAS inhibitors failed in clinical trials. PI3K inhibitors, for example, were ineffective at low acceptable doses, but large dosages or long-term therapy resulted in significant side effects and toxicities, as seen with LY294002 [17,172,173,174].

Significant efforts have been made to provide novel therapeutic technologies to dissect the complexity of GBM biology, as well as crossing the BBB [175,176]. Nevertheless, poor BBB permeability and intertumor heterogeneity remain the primary therapeutic challenges for GBM [13,160,167].

## 5. Tumor Heterogeneity

**Figure 4:**
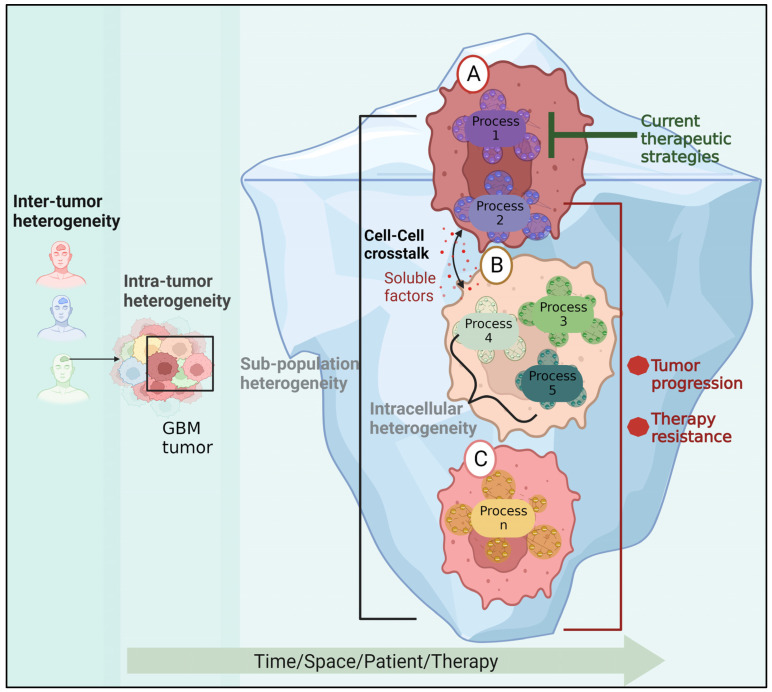
The role of GBM heterogeneity in GBM recurrence. In addition to patient variability, the cells within the tumor may belong to different subpopulations and carry a variety of active molecular processes. An untargeted process in cell (**A**) may trigger survival molecular mechanisms in cell (**B**) via soluble factors, resulting in the emergence of a new resistant subpopulation and tumor progression. Cell (**C**) represents another innately resistant subpopulation.

Inter-tumor heterogeneity is the term used to describe the heterogeneity among patients harboring tumors of various histological or molecular types. The existence of phenotypically and molecularly distinct cell populations within a tumor that exhibit varying degrees of resistance to existing treatments is known as intra-tumor heterogeneity (Figure 4). Using transcriptional profiling data from bulk tumor tissues, four subgroups of GBM tumors were identified: mesenchymal, classical, proneural, and neural. A recent study identified 18 driver genes, including *MGMT*, *ATRX*, *H3F3A*, *TP53*, *EGFR*, *NES*, *VIM*, *MIK67*, and *OLIG2*, with differential expression profiles in different molecular subtypes [177]. For instance, Herrera-Oropeza et al. found that the classical subtype showed overexpression of *EGFR*, *NES*, *VIM*, and *TP53*, while the proneural subtype was characterized by the overexpression of *MKi67* and *OLIG2*. The mesenchymal subtype showed the overexpression of *MGMT* and *VIM*, and the repression of *EGFR*, *H3F3Q*, *OLIG2*, *S100*, and *TP53*. In fact, it was discovered that *NES*, *OLIG2*, *VIM*, and *EGFR* were sufficient to subtype GBM into four subgroups, as confirmed by another investigation [178].

High-throughput proteomics studies, which quantify protein–protein expression patterns across a large sample of GBM tumors, show a great diversity among GBM patients, resulting in dozens of subgroups. Some patients do not fit into any groupings because they have specific changes in protein–protein expression networks [6,179].

Recent investigations showed that the expression of GBM biomarkers is not homogenous, indicating that there is not only variation across patients but also within the tumor cells [1]. Sottoriva et al. demonstrated that fragments from the same tumor can be categorized into several GBM subgroups by genomic analysis of samples from various locations of a single GBM tumor. In this regard, they discovered that one tumor clone exhibited *EGFR*, *CDK6*, and *MET* amplification, whereas another subclone received a copy of chromosome 3 with *PIK3CA*, resulting in *PIK3CA* amplification [7].

The existence of diverse subpopulations (Figure 4) underlies tumor plasticity, resulting in resistance [8] to RTK inhibitors [180] or radiotherapy [181]. Moreover, different regions within the tumor tissue can have varying degrees of radiosensitivity, as was demonstrated using patient-derived neurosphere cultures [182].

Furthermore, microenvironmental pressures like hypoxia, acidosis, and reactive oxygen species can arbitrarily cause genetic instability, resulting in the formation of de-novo therapy-resistant subpopulations [9]. Resolving and targeting the expanding cellular subpopulations in response to therapy, as has recently been demonstrated in other cancer types, may be an effective method for reducing drug resistance development [183].

### Cell–Cell Communication within GBM

Cell–cell communication between GBM subpopulations or between GBM cells and the cells in the tumor microenvironment is crucial for maintaining GBM development [96]. Factors released by microenvironment cells, like Chi3l1, or ligands, like IL-6 or HGF, that mediate the interaction of GBM subpopulations facilitate cell–cell contact [96,184], resulting in the generation and maintenance of a diversity of transcriptome and phenotypic states inside the tumor [185]. They contribute to the tumor’s aggressiveness by creating a cellular network through which cancer cells communicate with one another or other tumoral microenvironment components to stimulate their growth, invasion, neo-angiogenesis, oncogenic transformation, and immune suppression [186].

For instance, it was found that astrocytes interact closely with tumor cells, forming a network of communication crucial to tumor development [186]. Astrocytes secrete a large number of soluble factors that encourage GBM invasiveness and growth by activating several intracellular signaling pathways, such as NF-Kb and STAT1, in GBM cells [187,188,189]. As a result, GBM tumors inhibit the expression of p53 in astrocytes, promoting the survival of GBM cells through ECM remodeling [190]. Another study revealed that the communication between GBM cells and non-cancerous astrocytes drives tumor growth through mitochondrial transfer from astrocytes to GBM cells [191].

In the research on the GBM microenvironment, macrophages have also received a lot of interest since they comprise the majority and up to 30% of the tumoral mass [192]. Several signaling pathways involved in glioma invasion, including the TGF-B, EGF, and PDGF signaling pathways, were found to be stimulated by macrophages [193,194]. Another illustration of macrophage–tumor interaction is macrophage-dependent angiogenesis. IL-6 secretion, JAK-STAT activation, and increased Src-PI3K-YAP signaling all contribute to the process [195,196]. Furthermore, recent research showed that the crosstalk between GBM cells and macrophages promotes tumor growth and progression by evolving heterogeneous mechanisms that permit malignant glioma cells to enfeeble microglia and brain macrophage defense systems [197]. These mechanisms include various pathways, such as IL-6, IL-33, m-TOR, CCN4, miR-155-3p, and miR-1246 [197].

Additionally, communication between the tumor subpopulations is essential for the progression of GBM. It was discovered, using U87 model cell lines and GBM patient-derived cells, that paracrine interactions between the subpopulations expressing activating mutations (EGFRvIII) and subpopulations harboring epidermal growth factor receptor amplification (EGFRwt) play a significant role in the diffuse architectures of GBM tumors. It was shown that aggressive EGFRvIII cells alter the ability of EGFRwt cells to migrate and invade. HGF and IL6 released by EGFRvIII cells activate Src protein in EGFRwt cells, enhancing the EGFRwt cell-spreading ability and velocity [184]. Additionally, communication between these cell subtypes was found to support the tumor growth and heterogeneity of GBM [96].

## 6. Blood–Brain Barrier (BBB) in GBM

Although there is a growing list of the mechanisms underlying GBM development and resistance, and there is a high potential for using this knowledge to provide new therapeutic strategies, brain cancer medicine still needs to overcome an additional barrier to be able to implement potential therapeutic strategies. The blood–brain barrier (BBB) is a significant hurdle to the delivery of therapeutic drugs.

The BBB serves as a protection barrier between the circulatory system and the central nervous system’s extracellular space. The endothelial cells that make up the majority of the BBB, form a tight barrier along the blood vessel wall and regulate which substances can enter the parenchyma [198]. Thus, more than 98% of small molecules cannot penetrate tight junctions, which are smaller than 1 nm.

Although the BBB in GBM may have increased permeability as a result of poorly developed, leaky blood vessels, upregulated transporter proteins, and downregulated tight junction proteins [199,200], the disruption of the tumor’s BBB is not uniform, and almost all GBM patients have large tumor regions with an intact BBB [201].

In order to effectively treat GBM patients with drugs, several technologies are being developed [202,203]. For instance, chemical delivery systems (CDSs), which link an active drug molecule to a lipophilic carrier to boost a drug’s solubility and cell permeability or lipidation of the therapeutic molecule, are examples of such efforts [167].

Using polymeric nanoparticles (NPs) is another promising therapeutic method [204,205]. NPs are carriers with diameters ranging from 10 to 1000 nm that can be engineered from various materials, including metal-, lipid-, and polymer-based materials. They can be conjugated into multiple chemotherapeutic and targeted drugs. For instance, Alessandro Sacchetti et al. designed a gel formulation that enhances the release of TMZ locally beyond the BBB in orthotopic human xenograft models [206]. Another study suggested a novel design of flexibility-tunable polymer-drug conjugates to deliver drug combinations with ratiometric dosing and reported that focused ultrasound (FUS) improved the penetration of the drug conjugates into murine brain GBM models [207]. Furthermore, a clinical study was conducted on the use of an implantable ultrasound device, for delivering albumin-bound paclitaxel in patients with recurrent GBM [208]. The device could transiently open the BBB, allowing a safe and repeated penetration of cytotoxic drugs into the brain. Following these results, a phase 2 clinical trial is taking place to further evaluate the safety and efficacy of the approach and is registered with ClinicalTrials.gov (NCT04528680).

## 7. Computational Modeling of GBM: New Insights toward Understanding and Treating GBM

Computational GBM research is a rapidly growing multidisciplinary subject frequently utilized to investigate and define tumor heterogeneity; the effect of BBB constraints on treatment effectiveness; and GBM behavior, such as aggressiveness and recurrence.

For example, Randles et al. described the development of a spatially explicit stochastic process modeling to investigate the impact of the perivascular niche spatiotemporal dynamics in GBM, which can be used to optimize standard treatment (chemotherapy and radiation) schedules for this disease [209]. Another study proposed a model informed by in silico signaling pathways and kinetics characteristics to predict outcomes and prescribe customized therapy in GBM patients treated with radiation and TMZ [210].

Partial least-squares regression (PLSR) data-driven models that were constructed based on the paired signaling and phenotype data to predict the efficacy of phosphatase inhibition in GBM treatment [211] or to identify new targetable GBM markers are additional examples [212].

Information-theoretical approaches were used [6,179] to identify patient-specific signaling signatures in each GBM patient. Based on these signatures, patient-specific targeted drug combinations can be designed. This method was verified for other types of cancer [183,213].

Multiple machine learning (ML)/deep learning approaches have been extensively used to identify new potential therapeutic targets (reviewed in [214]). For example, based on progression-free survival (PFS), ML-based models (random forest classifier (RFD), extreme gradient boosting (XGBoost), naïve Bayes, and support vector machine (SVM)), algorithms were created to stratify newly diagnosed GBM patients into prognostic subclasses, identifying those at increased risk of early recurrence [215,216]. Wang et al. used machine learning algorithms (least absolute shrinkage and selection operator (LASSO) regression, SVM, RFB, and XGBoost) to identify individuals who may react better to immunotherapy and have higher overall survival [217].

Further examples include mathematical and bioinformatics models that attempt to predict BBB permeability and drug delivery efficacy [218,219]. For example, based on experimental information from preclinical subjects treated with anti-EGFR targeted therapy, an ordinary differential equation (ODE) model was developed to characterize the heterogeneous sensitivity of drug response and blood–brain barrier penetration [220].

Another study created a geometrical model that employed computational fluid dynamics to predict blood flow behavior with injected magnetic nanoparticles under various conditions, such as blood flow fluctuations. This provided information on the permeability of the BBB [221]. One more study used three-compartment cellular modeling (apical, cell monolayer, and basolateral) and statistical approaches to successfully simulate the time course of drug cellular uptake and accumulation, based on its BBB passive permeability, in order to demonstrate the functional relevance of uptake and efflux transporters to BBB penetration of drugs [222].

Overall, computational and theoretical models provide important insights into GBM heterogeneity, drug permeability across the BBB, tumor growth, and treatment responses, hence improving the efficacy of GBM dissection and individualized therapies.

## 8. Conclusions

Despite several advances in developing effective treatment regimens, GBM is incurable. The standard course of treatment includes radiotherapy, chemotherapy, and surgery. However, most patients experience tumor relapse and recurrence. From a therapeutic standpoint, invasion, intra- and inter-tumor heterogeneity, and BBB pose significant barriers to curative treatment.

The current primary challenge is to develop computational and experimental approaches for designing individualized multimodal treatments. The therapy should target ongoing patient-specific processes responsible for GBM infiltration and drug resistance. The proposed therapy should also consider basal or evolving states in response to treatment, as well as the interaction between the microenvironment and GBM, and should be designed to effectively transport a drug cocktail via the BBB.

## Abbreviations

GBM	Glioblastoma
CNS	Central nervous system
TMZ	Temozolomide
BBB	Blood–brain barrier
IDH	Isocitrate Dehydrogenase 1
NOS	Nitric Oxide Synthase
EMT	Epithelial–mesenchymal transition
ECM	Extracellular matrix
MMPs	Matrix Metalloproteinases
GTR	Gross total resection
MGMT	O6 Methylguanine DNA Methyltransferase
GSCs	GBM stem cells
EMT	Epithelial–mesenchymal transition
CAR T	Chimeric antigen receptor T-cells
TGF-β	Transforming Growth Factor Beta
Cx43	Connexin43
RT	Radiotherapy
P53	Tumor Protein 53
ATM gene	Ataxia-Telangiesctasia Mutated
MDM2	Mouse Double Minute 2
CSC	Cancer Stem Cell
PTEN	Phosphatase and Tensin Homolog
Akt	Protein Kinase B
PI3K	Phosphoinositide 3-Kinases
TTFs	Tumor-treating fields
EEF1D	Elongation Factor 1-Delta
MICAL2	Calponin and LIM Domain Containing 2
p-Smad2	Mothers against decapentaplegic homolog 2
ACTN1	Alpha-Actinin-3
CCND1	Cyclin D1
HCLS1	Hematopoietic Cell-Specific Lyn Substrate 1
ITGB5	Integrin Subunit Beta 5
PFN2	Profilin 2
PTPRC	Protein Tyrosine Phosphatase Receptor Type C
Gy	Gray (unit)
MAPK	Mitogen-Activated Protein Kinases
EGFR	Epidermal Growth Factor Receptor
EGF	Epidermal Growth Factor
VEGFR	Vascular Endothelial Growth Factor Receptor
FGFR	Fibroblast Growth Factor Receptor
PDGFR	Platelet-derived Growth Factor Receptor
SOX2	SRY-Box Transcription Factor 2
HGFR	Hepatocyte Growth Factor Receptor
IGFR	1R-Insulin-like growth factor receptor 1
DDR	Discoidin Domain Receptor
TME	Tumor microenvironment
RTK	Receptor tyrosine kinase
DDR	DNA damage response
TKI	Tyrosine Kinase Inhibitor
RTK	Receptor tyrosine kinases
NES	Nestin
OLIG2	Oligodendrocyte Transcription Factor
VIM	Vimentin
Il-6	Interleukin-6
Chi3l1	Chitinase 3 Like 1
NF-Kb	Nuclear Factor Kappa Light Chain Enhancer of Activated B Cells
STAT1	Signal Transducer and Activator of Transcription 1
JAK-STAT	Janus kinase/signal transducers and activators of transcription
ATRX	Alpha Thalassemia X-linked
H3F3A	Histone H3.3A
H3F3Q	Histone H3.3Q
MIK67	Marker of Proliferation Ki-67
mTOR	Mammalian Target of Rapamycin
IL-33	Interleukin 33
CCN4	Cellular Communication Network Factor 4
MiR	MicroRNA
YAP	Yes-Associated Protein 1
CDS	Chemical delivery system
NPs	Polymeric nanoparticles
FUS	Focused ultrasound
PLSR	Partial least-squares regression
ML	Machine learning
XGBoost	eXtreme gradient boosting
SVM	Support vector machine
LASSO	Least absolute shrinkage and selection operator
RFB	Receptive field block
